# Differences in Hospital Costs among Octogenarians and Nonagenarians Following Primary Total Joint Arthroplasty

**DOI:** 10.3390/geriatrics6010026

**Published:** 2021-03-09

**Authors:** Christopher Fang, Andrew Hagar, Matthew Gordon, Carl T. Talmo, David A. Mattingly, Eric L. Smith

**Affiliations:** 1 New England Baptist Hospital, 125 Parker Hill Ave, Boston, MA 02120, USA; cfang@nebh.org (C.F.); ctalmo@nebh.org (C.T.T.); dmatting@nebh.org (D.A.M.); 2 Tufts Medical Center, Department of Orthopaedic Surgery, 800 Washington St, Boston, MA 02111, USA; ahagar@tuftsmedicalcenter.org (A.H.); mgordon1@tuftsmedicalcenter.org (M.G.)

**Keywords:** older patients, total hip arthroplasty, total knee arthroplasty, octogenarians, nonagenarians, TDABC, costs

## Abstract

The proportion of patients over the age of 90 years continues to grow, and the anticipated demand for total joint arthroplasty (TJA) in this population is expected to rise concomitantly. As the country shifts to alternative reimbursement models, data regarding hospital expenses is needed for accurate risk-adjusted stratification. The aim of this study was to compare total in-hospital costs following primary TJA in octogenarians and nonagenarians, and to determine the primary drivers of cost. This was a retrospective analysis from a single institution in the U.S. We used time-drive activity-based costing (TDABC) to capture granular total hospital costs for each patient. 889 TJA’s were included in the study, with 841 octogenarians and 48 nonagenarians. Nonagenarians were more likely to undergo total hip arthroplasty (THA) (70.8% vs. 42.4%; *p* < 0.0001), had higher ASA classification (2.6 vs. 2.4; *p* = 0.049), and were more often privately insured (35.4% vs. 27.8%; *p* = 0.0001) as compared to octogenarians. Nonagenarians were more often discharged to skilled nursing facilities (56.2% vs. 37.5%; *p* = 0.0011), experienced longer operating room (OR) time (142 vs. 133; *p* = 0.0201) and length of stay (3.7 vs. 3.1; *p* = 0.0003), and had higher implant and total in-hospital costs (*p* < 0.0001 and 0.0001). Multivariate linear regression showed implant cost (0.700; *p* < 0.0001), length of stay (0.546; *p* < 0.0001), and OR time (0.288; *p* < 0.0001) to be the strongest associations with overall costs. Primary TJA for nonagenarians was more expensive than octogenarians. Targeting implant costs, length of stay, and OR time can reduce costs for nonagenarians in order to provide cost-effective value-based care.

## 1. Introduction

The age of the population in the United States is increasing. By 2030, all baby boomers will be older than 65 years of age, and by 2060, the population is expected to grow by 79 million people [1]. Between 2010 and 2050, the number of people 90 years of age or older is expected to quadruple [2]. The anticipated case load of total joint arthroplasty (TJA) in these oldest of patients will likely grow as the prevalence of degenerative joint disease increases in these populations. Further, nonoperative management of osteoarthritis in these older patients may be contraindicated due to their inability to tolerate non-steroidal anti-inflammatory drugs as a result of an associated medical comorbidity such as renal disease or gastrointestinal intolerance [3].

Clinical outcomes for total hip arthroplasty (THA) and total knee arthroplasty (TKA) in nonagenarian patients have been shown to be excellent [4]; however, when compared to octogenarians undergoing TJA, nonagenarians had significantly higher inpatient mortality and complication rates [5,6]. As there are differences for outcomes between these two age groups, it is likely there are differences in the costs of these procedures.

Therefore, it is important to understand how an increase in comorbidities and the rate of complications may implicate hospital costs, and what other potential expenditures may impact the total cost differences between these populations.

Our aim is to determine the differences in costs for a TJA episode of care for nonagenarians as compared with octogenarians, and what factors contribute to a higher total hospital cost. Our hypothesis is that nonagenarians will have increased hospital costs for TJA compared to octogenarians. All members of the healthcare system can use this information to effectively focus on drivers of cost to mitigate expenses for these vulnerable patients.

## 2. Materials and Methods

### 2.1. Study Design

We retrospectively analyzed data between January 2018 through May 2020 at our single-specialty orthopaedic hospital to gather patients over 80 years of age who underwent either primary THA or TKA. We gathered data including age, American Society of Anesthesiologists (ASA) classification, procedure type, insurance, discharge disposition, operating room (OR) time, length of stay, implant cost and total in-hospital cost, from our de-identified cost-analysis patient registry to compare differences between octogenarians and nonagenarians. Financial data is presented as indexed values to protect institutional proprietary information.

### 2.2. Time-Driven Activity-Based Costing

Cost information for the study was determined with the use of a third-party, commercial medical cost-analysis database, Avant-Garde Health (Boston, MA, USA). Our institution’s billing records are sourced to the database, where all information is de-identified and maintained. Time-driven activity-based costing (TDABC) is a cost-accounting methodology that was designed to improve value [7], and was used to determine all costs at our institution for the procedures. TDABC provides granular patient cost data based on total personnel and supply costs used directly by the patient during their episode of care to calculate total costs. Itemized costs for implant and total in-hospital expenses were collected for each patient. Indirect costs not specific to the patient (e.g., administrative fees) were not included in this study.

### 2.3. Statistical Analysis

To compare the characteristics and outcomes of octogenarians and nonagenarians undergoing THA and TKA, chi-square and *t*-tests were used where appropriate. Multivariate linear regression was used to estimate the relationship between total in-hospital cost and other significant independent variables such as OR time, implant cost, and length of stay. A standardized beta coefficient was used to compare the strength of the effect of each individual independent variable to total in-hospital costs. All statistical analyses were performed using SAS v9.4 (SAS Institute, Cary, NC, USA). Significance was set at 0.05.

Because this study only included deidentified, Health Insurance Portability, and Accountability Act-compliant data from our cost-analysis database without primary data, it was exempt from institutional review board approval.

## 3. Results

Eight hundred and eighty-nine primary TJA’s were included in the study (Table 1). Of these procedures, 841 were performed on octogenarian patients and 48 on nonagenarian patients (Table 1). Nonagenarians on average had a higher ASA classification (2.6 vs. 2.4; *p* < 0.0001), underwent more THAs (70.8% vs. 42.4%; *p* = 0.0001), and less frequently had Medicare (64.6% vs. 72.2%; *p* = 0.0001) (Table 1).

### 3.1. THA

Three hundred and ninety-one (44%) of the procedures were THAs. Among THAs, nonagenarians were more often discharged to skilled nursing facilities (SNF) (56% vs. 37.7%; *p* = 0.0054), experienced longer length of stay (3.5 vs. 3; *p* = 0.0168), and had 7% higher implant and total in-hospital costs (*p* = 0.0488 and 0.0071, respectively) (Table 2). Nonagenarians and octogenarians did not differ in OR time for THA.

### 3.2. TKA

Four hundred and ninety-eight (56%) of the procedures were TKAs. Among TKAs, nonagenarians experienced longer length of stay (4.3 vs. 3.2; *p* = 0.0003) and had 33% higher implant and 25% higher total in-hospital costs (*p* < 0.0001 and 0.0001, respectively) (Table 3). Nonagenarians and octogenarians did not differ in discharge position or OR time for TKA.

### 3.3. THA and TKA

Overall, nonagenarians were more often discharged to skilled nursing facilities (SNF) (56.2% vs. 37.5%; *p* = 0.0011), experienced longer OR time (142 vs. 133; *p* = 0.0201) and length of stay (3.7 vs. 3.1; *p* = 0.0003), and had 23% higher implant and 14% higher total in-hospital costs (*p* < 0.0001 and 0.0001, respectively) (Table 4).

Table 5 reports the results from the multivariate linear regressions analysis on total in-hospital costs. The independent variables explained 97% of the variation in hip and knee TJA in-hospital costs (Table 5). The implant cost was associated with the strongest effect (0.700; *p* < 0.0001) on total in-hospital cost (Table 5). Length of stay was associated with the second strongest effect (0.546; *p* < 0.001), and OR time was associated with the third strongest effect (0.288; *p* < 0.001) on total in-hospital cost (Table 5). THA (−0.101; *p* < 0.001) and Medicare insurance (−0.025; *p* < 0.001) were associated with a decrease in total in-hospital costs (Table 5). The nonagenarian group had a positive effect on total in-hospital costs that was consistent with our hypothesis (0.004), but it was not statistically significant at the 5% level, meaning there was more than 5% probability that the true value of this effect was not different from zero.

## 4. Discussion

By 2050, the population of persons 90 years or older will reach 10% in the United States [2]. With this shift in population age, prevalence of osteoarthritis and TJA will increase among octogenarians and nonagenarians. Building on prior studies that looked at the outcomes and morbidity of these older patients, our study compared overall costs for TJA and showed that implant and in-hospital costs for nonagenarians were more expensive than octogenarians. Value in healthcare is described as the health outcomes achieved per dollar spent, as described by Porter [8]. In order to best provide value-based health care (VBHC), it is important to understand the costs associated with TJA procedures between patient populations. After identifying disparities among patient groups, drivers of cost can then be targeted to decrease overall health expenses.

In our study, we found that nonagenarians had a higher ASA classification than octogenarians. ASA ratings correlate with patient medical comorbidities and can be helpful in predicting perioperative risks and outcomes. Previous studies have looked at outcomes for nonagenarians and octogenarians [4,5,6,9]. For both TKA and THA, nonagenarians more frequently required blood transfusions, and developed sepsis, urinary tract infections, and acute kidney injuries more often than octogenarians [5,6]. For primary TKA, nonagenarians were found to have a higher inpatient mortality rate than octogenarians [5]. For primary THA, nonagenarians were not found to have a significantly higher inpatient mortality rate than octogenarians [6]. While nonagenarians were found to have a higher risk of in-hospital mortality compared to younger patients for both THA and TKA [9], the data suggests mortality between nonagenarians and octogenarians is the same for THA. This decreased risk in mortality could potentially explain why nonagenarians more often underwent THA than TKA in our study.

Privately insured patients have been shown to have fewer medical complications and lower mortality rates when compared with Medicare patients undergoing primary THA and TKA [10,11]. In our study, nonagenarians were more often privately insured than octogenarians. This could be due to a variety of reasons, including increasingly elderly patients employing private insurance to help cover services not covered by Medicare. Future research investigating the discrepancy on outcomes and costs between insurance payers is needed to optimize resource-allocation among elderly patients undergoing TJA.

Previous studies examining costs of TJA have shown nonagenarians to have significantly higher total hospital charges as compared to younger patients [9]. Compared to octogenarians, nonagenarians were also shown to have significantly higher total charges for TKA and THA [5,6]. However, charges do not represent actual costs of care. Charges are the individual list price a hospital sets for claim purposes, and neither Medicare nor most private insurers pay the full charge price. Thus, charges are a suboptimal measure to determine the actual expenses incurred by an institution for providing the care associated with the procedure. Our study, as determined by TDABC, a modern, validated, and more accurate cost methodology for primary TJA [12,13,14], showed significantly higher episode of care costs for TKA and THA among nonagenarians than octogenarians.

Nonagenarians are at a higher risk of developing complications, which may be reflected in their significantly higher length of stay to manage these complexities. With TDABC, the increased personnel and time is accurately captured to provide granular cost data. This increases the sensitivity to capture differences in costs incurred by the hospital treating nonagenarians and octogenarians. Additionally, nonagenarians had significantly longer OR time, higher implant costs, and were more often discharged to inpatient rehab or skilled nursing facilities as compared to octogenarians. All of these factors contributed to a higher total in-hospital cost. In order to measure the relative effect of these variables, we performed a multivariate linear regression model with standardized estimates. Implant costs were shown to be the largest driver of in-hospital total costs for our study. This is consistent with the literature regarding implant costs as the major driver of overall costs for primary TJA regardless of age [15].

Beyond implant costs, length of stay and OR time were the most significant factors on total in-hospital costs. As mentioned previously, nonagenarians are at higher risk for developing in-hospital complications such as urinary tract infections and acute kidney injuries, which could increase their length of stay. The increased OR time may reflect the added complexity to surgical cases that the eldest patients present. The impact of nonagenarian versus octogenarian status was small in magnitude and not statistically significant after compensating for the effects of holding all other significant variables constant. This may be explained by the relatively large association of implant costs, length of stay, and OR time on overall hospital costs. These findings suggest that nonagenarian vs. octogenarian status may not strictly be considered an independent risk factor for increased hospital costs when stratifying patients for TJA. However, because octogenarians and nonagenarians are at an increased risk for complications that directly influence these variables, added efforts should focus on decreasing implant costs, length of stay, and OR time to reduce overall costs for all patients over 80 years of age.

Strengths of this study include a relatively large sample size of 889 TJA procedures for patients greater than the age of 80 years over a two-year period. Further, our methodology of costing using TDABC has been shown to be more accurate for primary TJA than hospital charges or traditional accounting [12,13]. The current study contains several limitations, including its retrospective nature. The analysis of episode of care costs were dependent on accurate administrative coding for TDABC process maps. Our sample size for nonagenarians is only 48; however, this limited patient group represents a scarce study population, and our sample size is relatively large in comparison to a previous retrospective study of 39 nonagenarian patients [4]. The nonagenarian group also underwent a significantly higher amount of THA procedures, which is more expensive than TKA for the general population at our institution. However, our multivariate regression analysis showed that THA procedures actually decreased total in-hospital costs when controlling for all other variables. Thus, a higher percentage of THA procedures does not completely explain why TJA for nonagenarians were more expensive than octogenarians. The sample size was comprised of a single orthopaedic institution, which limits the generalizability of the results to other hospitals, such as tertiary centers.

## 5. Conclusions

In conclusion, overall hospital costs for primary TJA were more expensive for nonagenarians than octogenarians. As our population ages, we must prepare for the imminent demand for TJA in the oldest of patients. Higher implant costs were seen to be a primary driver of total costs, along with a higher length of stay, longer OR time, insurance, discharge disposition, and procedure type. This information is valuable to all members of the healthcare system as the country develops reimbursement models for TJA. As we shift to deliver value-based health care with a focus on outcomes per dollars, identifying drivers of cost for this vulnerable patient population is fundamental to providing cost-effective care.

## Figures and Tables

**Table 1:** Comparison of patient characteristics for octogenarians and nonagenarians undergoing primary total hip or knee arthroplasty. ASA = American Society of Anesthesiologists classification.

	Octogenarians (*n* = 841)	Nonagenarians (*n* = 48)	*p*-Value
Age (years), mean (SD)	82.9 (2.4)	91.5 (1.4)	<0.0001
ASA	2.4 (0.5)	2.6 (0.5)	0.049
Procedure type			0.0001
Hip	42.4%	70.8%	
Knee	57.6%	29.2%	
Insurance			0.0001
Medicare	72.2%	64.6%	
Private	27.8%	35.4%	

**Table 2:** Comparing outcomes and costs for total hip arthroplasty between octogenarians and nonagenarians. SNF = skilled nursing facility. OR = operating room.

	Octogenarians (*n* = 357)	Nonagenarians (*n* = 34)	*p*-Value
Discharge Disposition			0.0054
Home	49.2%	20.5%	
Inpatient Rehab	13.1%	23.5%	
SNF	37.7%	56.0%	
OR Time (minutes)	138.2 (27)	142.9 (40.4)	0.3574
Length of Stay (days)	3.0 (1.0)	3.5 (1.1)	0.0168
Implant Cost	$ --	+7%	0.0488
Total In-Hospital Cost	$ --	+7%	0.0071

**Table 3:** Comparing outcomes and costs for total knee arthroplasty between octogenarians and nonagenarians. SNF = skilled nursing facility. OR = operating room.

	Octogenarians (*n* = 484)	Nonagenarians (*n* = 14)	*p*-Value
Discharge Disposition			0.2421
Home	51.0%	28.5%	
Inpatient Rehab	11.9%	14.2%	
SNF	37.1%	57.3%	
OR Time (minutes)	129.2 (23.5)	139.9 (23.5)	0.0938
Length of Stay (days)	3.2 (1.0)	4.3 (2.6)	0.0003
Implant Cost	$ --	+33%	<0.0001
Total In-Hospital Cost	$ --	+25%	<0.0001

**Table 4:** Comparing outcomes and costs for total hip and knee arthroplasty between octogenarians and nonagenarians. SNF = skilled nursing facility. OR = operating room.

	Octogenarians (*n* = 841)	Nonagenarians (*n* = 48)	*p*-Value
Discharge Disposition			0.0011
Home	50.2%	22.9%	
Inpatient Rehab	12.3%	20.9%	
SNF	37.5%	56.2%	
OR Time (minutes)	133 (25.4)	142 (36.1)	0.0201
Length of Stay (days)	3.1 (1.0)	3.7 (1.6)	0.0003
Implant Cost	$ --	+23%	<0.0001
Total In-Hospital Cost	$ --	+14%	<0.0001

**Table 5:** Multivariate linear regression for total in-hospital cost. Adjusted R-square = 0.9713. OR = operating room.

Parameter	Standardized Estimate	95% Confidence Limits		*p* Value
Implant Cost	0.700	0.997	1.039	<0.0001
OR Time (minutes)	0.288	13.826	15.046	<0.0001
Length of Stay (days)	0.546	644.531	673.441	<0.0001
Age GroupNonagenarian	0.004	−45.193	88.327	0.5262
Procedure TypeHip	−0.101	−302.116	−231.715	<0.0001
Insurance Medicare	−0.025	−105.339	−39.778	<0.0001
Discharge Disposition Home	0.015	7.023	73.395	0.0176
Discharge Disposition Inpatient Rehab	0.006	−24.467	70.475	0.3418

## Data Availability

Data sharing is not applicable to this article due to proprietary financial restrictions.
